# *Bifidobacterium breve* and *Bifidobacterium longum* Attenuate Choline-Induced Plasma Trimethylamine N-Oxide Production by Modulating Gut Microbiota in Mice

**DOI:** 10.3390/nu14061222

**Published:** 2022-03-14

**Authors:** Qianqian Wang, Min Guo, Yang Liu, Mengshu Xu, Liuting Shi, Xiu Li, Jianxin Zhao, Hao Zhang, Gang Wang, Wei Chen

**Affiliations:** 1 State Key Laboratory of Food Science and Technology, Jiangnan University, Wuxi 214122, China; 7160112022@stu.jiangnan.edu.cn (Q.W.); guomin@jiangnan.edu.cn (M.G.); 6190112118@stu.jiangnan.edu.cn (M.X.); 1012180809@stu.jiangnan.edu.cn (L.S.); lixiu@jiangnan.edu.cn (X.L.); zhaojianxin@jiangnan.edu.cn (J.Z.); zhanghao61@jiangnan.edu.cn (H.Z.); chenwei66@jiangnan.edu.cn (W.C.); 2 School of Food Science and Technology, Jiangnan University, Wuxi 214122, China; 3 KLATASDS-MOE, School of Statistics, East China Normal University, Shanghai 200062, China; yliu@sfs.ecnu.edu.cn; 4 (Yangzhou) Institute of Food Biotechnology, Jiangnan University, Yangzhou 225004, China; 5 National Engineering Research Center for Functional Food, Jiangnan University, Wuxi 214122, China; 6 Wuxi Translational Medicine Research Center, Jiangsu Translational Medicine Research Institute Wuxi Branch, Wuxi 214122, China

**Keywords:** probiotics, choline, atherosclerosis, trimethylamine N-oxide, trimethylamine, gut microbiota

## Abstract

Atherosclerosis is the main cause of myocardial infarction and stroke, and the morbidity and mortality rates of cardiovascular disease are among the highest of any disease worldwide. Excessive plasma trimethylamine-N-oxide (TMAO), an intestinal metabolite, promotes the development of atherosclerosis. Therefore, effective measures for reducing plasma TMAO production can contribute to preventing atherosclerosis. Probiotics are living microorganisms that are beneficial to the human body, and some of them can attenuate plasma TMAO production. To explore the effects of probiotic supplementation on plasma TMAO in choline-fed mice, we intragastrically administered eight strains of *Bifidobacterium breve* and eight strains of *Bifidobacterium longum* to mice for 6 weeks. *B. breve* Bb4 and *B. longum* BL1 and BL7 significantly reduced plasma TMAO and plasma and cecal trimethylamine concentrations. However, hepatic flavin monooxygenase (FMO) activity, flavin-containing monooxygenase 3 (FMO3), farnesoid X receptor (FXR) protein expression and TMAO fractional excretion were not significantly affected by *Bifidobacterium* supplementation. The treatment of *Bifidobacterium* strains modulated the abundances of several genera such as *Ruminococcaceae* UCG-009, *Ruminococcaceae* UCG-010, which belong to the Firmicutes that has been reported with *cut* gene clusters, which may be related to the reduction in intestinal TMA and plasma TMAO. Additionally, a reduction in *Ruminococcaceae* indicates a reduction in circulating glucose and lipids, which may be another pathway by which *Bifidobacterium* strains reduce the risk of atherosclerosis. The effect of *Bifidobacterium* strains on *Bacteroides* also suggests a relationship between the abundance of this genus and TMA concentrations in the gut. Therefore, the mechanism underlying these changes might be gut microbiota regulation. These *Bifidobacterium* strains may have therapeutic potential for alleviating TMAO-related diseases.

## 1. Introduction

Cardiovascular diseases have the highest mortality and morbidity rates in the world, for which atherosclerosis (AS) is the main pathologic basis. AS occurrence and development are related to vascular inflammation, hypercholesterolemia and oxidative stress [1]. In recent years, researchers have found that gut microbiota play a key role in the occurrence and development of AS. High plasma trimethylamine-N-oxide (TMAO) concentrations, mainly from choline metabolite by gut microbiota or from fish intake, have been found to be an independent risk factor for promoting AS [2,3]. In humans, plasma TMAO is predominately generated from dietary nutrients containing trimethylamine (TMA) groups, such as phosphatidylcholine, choline and L-carnitine [4]. TMAO generation is regulated by gut microbiota, which metabolize dietary choline and L-carnitine to TMA. After TMA enters the liver through the portal vein, it is further metabolized to TMAO by hepatic flavin monooxygenases (FMOs) [5].

TMAO promotes AS occurrence and development in several ways. TMAO induces a vascular inflammatory response and inflammatory gene expression in the mouse aorta via activation of the mitogen-activated protein kinase (MAPK)/extracellular signal-regulated kinase/nuclear factor-κB signaling pathway [6], which stimulates the formation of NOD-like receptor protein 3 inflammatory bodies in the aorta and vascular endothelial cells, and induces interleukin-1β production [7,8]. TMAO promotes the formation of foam cells via cluster of differentiation 36/MAPK/c-Jun N-terminal kinase pathway modulation [9] and also disrupts the redox balance by inhibiting antioxidant enzymes such as superoxide dismutase (SOD) and catalase (CAT), thereby weakening the vascular antioxidant abilities of the aorta [8,10,11]. Moreover, TMAO promotes reactive oxygen species (ROS) production, creating vascular oxidative stress [8,10]. TMAO is also involved in cholesterol metabolism and bile acid metabolism in that it reduces reverse cholesterol transport and bile acid synthesis [5]. Conjugated bile acids are synthesized by liver cholesterol and, in the presence of intestinal microorganisms, are hydrolyzed to unconjugated bile acids. Unconjugated bile acids are hydrophobic and more easily excreted in feces [12]. Bile acid synthesis not only reduces cholesterol levels in the body but also regulates glucose homeostasis, energy expenditure and insulin resistance, which decrease AS risk [13].

Given the role of gut microbiota in the pathogenesis of TMAO-induced AS, gut microbiota offer a potential therapeutic target for preventing and treating cardiovascular disease. Dietary composition affects the community structure of intestinal microorganisms and gut microbiota quickly adapt to dietary changes. Ingesting dietary components that reduce TMAO production may be a promising strategy by which to prevent AS. In the light of growing evidence of the relationship between gut microbiota and chronic diseases, probiotics have received increasing attention as intervention targets. Probiotics are living microorganisms that are beneficial to humans and animals when administered in adequate amounts, and are mainly found in fermented foods, dairy products or human feces [14]. Probiotics show specificity and functionality for alleviating chronic diseases and reducing inflammation, and preclinical studies have been demonstrated to relieve liver damage [15], reduce weight [16] and remit colitis [17]. Previous research has already indicated that bifidobacteria were negatively correlated with plasma TMAO and TMA [18]. Boutagy et al. reported that supplementing multi strain probiotic VSL#3 containing bifidobacteria could reduce the concentration of TMAO induced by a high-fat diet [19]. Members of the genus *Bifidobacterium* have been identified as almost ubiquitous inhabitants of the human host. In this study, *B. breve* and *B. longum*, which are the predominant *Bifidobacterium* in infants and adults, respectively, were chosen to treat choline fed C57BL/6J mice to determine whether these two species of *Bifidobacterium* have effects on plasma TMAO levels [20,21].

## 2. Materials and Methods

### 2.1. Probiotic Strains and Culture

Eight strains of *B. breve* and eight strains of *B. longum* were isolated from the feces of healthy people and preserved in the Strain Preservation Center of Jiangnan University (Supplementary Materials Table S1). Some of these strains have been studied and published [22,23,24]. The strains were cultured with MRS medium containing 0.05% cysteine in anaerobic workstations. Methods for anaerobic culture, and preparation of bacterial suspension for gavage are described in detail in Supplementary Materials. The experiment did not involve human experiments, and the fecal samples of the isolated strains came from healthy volunteers and did not cause any foreseeable risk or discomfort to the participants. The volunteers signed a written informed consent form or obtained the consent of their legal guardian.

### 2.2. Animals and Treatment

In order to avoid difficulties in later analysis due to the low expression of FMO_3_ by androgens in male mice, female mice were considered for this study [4]. Seven-week-old female C57BL/6J mice were purchased from Beijing Vital River Laboratory Animal Technology Co., Ltd. Standard control feed (LAD3001M, 0.1% choline content) and 1.0% choline feed (LAD3001M, 1.0% choline content) were purchased from TROPHIC Animal Feed High-Tech Co. Ltd. (Nan Tong, China). All animal experiments were reviewed and approved by the Jiangnan University Ethics Committee (JN. No 20190915c1461230[225]) and conducted in the Experimental Animal Centre. Mice were housed in individually ventilated cages maintained at 25 ± 2 °C and 50 ± 5% humidity under a 12 h light/dark cycle. Food and water were provided ad libitum.

#### 2.2.1. Collected of Plasma after Intragastric Choline Chloride Administration

**Figure 1:**
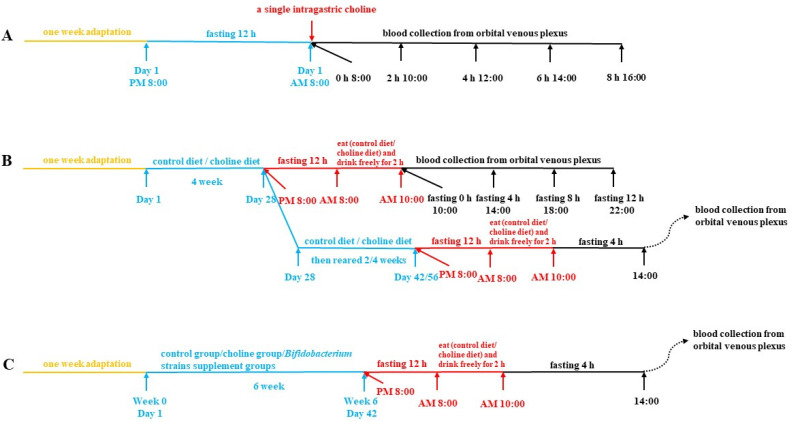
Timeline of experimental procedures used in this study: (**A**) collection of plasma after intragastric choline chloride administration, (**B**) collection of plasma after fasting, (**C**) collection of plasma after *B. breve* and *B. longum* supplementation.

After one week’s adaptation, a single intragastric dose of 400 mg choline chloride (Sigma-Aldrich) per kilogram of body weight was administered to seven-week-old healthy female C57BL/6J mice (*n* = 12) after fasting overnight. Blood was collected from the orbital venous plexus by capillary at 0 h, 2 h, 4 h, 6 h and 8 h after intragastric administration and placed in an anticoagulant centrifuge (Figure 1A). Plasma was separated via centrifugation (3500× *g*, 15 min, 4 °C) and stored at −80 °C [18].

#### 2.2.2. Collection of Plasma after Fasting

Seven-week-old healthy female C57BL/6J mice (*n* = 16) were divided into two diet groups based on body weight, after one week’s adaptation: (1) control diet and (2) 1.0% choline diet. After freely feeding for 28 days and fasting overnight, at 8:00 a.m. mice were allowed to eat and drink freely for 2 h before they were transferred to new cages without food and water. Blood was collected from the orbital venous plexus at 0 h, 4 h, 8 h and 12 h after transfer, and plasma was separated via centrifugation (3500× *g*, 15 min, 4 °C) and stored at −80 °C. Using the same method, plasma was collected after 4 h of fasting at 42 days and 56 days and stored at −80 °C (Figure 1B).

#### 2.2.3. Collection of Biological Samples after *B. breve* and *B. longum* Supplementation

Seven-week-old healthy female C57BL/6J mice (*n* = 108) were divided into 18 groups based on body weight, after one week’s adaptation: (1) control group (standard diet + 0.2 mL normal saline), (2) choline treatment group (1.0% choline diet + 0.2 mL normal saline), (3–10) eight *B. breve* strains supplement groups (1.0% choline diet + 0.2 mL Bb1–8, containing approximately 1 × 10^9^ colony-forming units (CFU)/mL), (11–18) eight *B. longum* strains supplement groups (1.0% choline diet + 0.2 mL BL1–8, containing approximately 1 × 10^9^ CFU/mL). Saline and suspensions were administered by gavage once daily for 6 weeks.

During the trial, mice were housed in clean cages and weighed weekly. Using the method in Section 2.2.2, plasma was collected after 4 h of fasting at 42 days, and stored at −80 °C (Figure 1C). It should be mentioned that, in order to monitor the changes of plasma TMAO of mice during feeding, 20 μL plasma was required to detect TMAO levels by LC-MS. From week 3, after 4 h of fasting, plasma was prepared according to the method above once a week. Before the end of the experiment, the mouse urine was continuously collected for 24 h with metabolic chambers and stored at −80 °C. Mice were allowed to eat and drink ad libitum during urine collection. At the end of the experiment, mice were anaesthetized using isoflurane, and plasma and tissue samples (liver, kidney, spleen, cecum) were collected. Before being snap-frozen in liquid nitrogen and stored at −80 °C, organ (liver, kidney, spleen) indexes were calculated. The organ index was defined as the ratio of organ weight to body weight. 

### 2.3. Determination of Biochemical Parameters of Plasma and Liver in Mice

The plasma levels of total cholesterol (TC), triacylglycerols (TG), high-density lipoprotein cholesterol (HDL-C), low-density lipoprotein cholesterol (LDL-C), aspartate aminotransferase (AST), and alanine aminotransferase (ALT) were measured by auto-biochemical analyzer. The protein expression of liver flavin-containing monooxygenase 3 (FMO3) and farnesoid X receptor (FXR) were determined by commercial Elisa kits purchased from Shanghai Enzyme-linked Biotechnology Institute (Shanghai, China). The protein levels of cecum glycyl radical enzyme (CutC) and glycyl radical-activating protein (CutD) were determined by commercial Elisa kits purchased from Wuhan Mosak Biotechnology Co., Ltd. (Wuhan, China).

### 2.4. Quantification of TMAO, TMA and d9-TMAO Levels

#### 2.4.1. Plasma Sample Preparation

The 20 μL plasma sample was added to 80 μL acetonitrile to precipitate the protein, and d9-TMAO with the final concentration of 2.0 μM was added to the plasma sample as the internal standard. After the mixture was fully mixed, it was placed at −80 °C for 2 h and centrifuged for 15 min at 12,000× *g* at 4 °C. The supernatant was transferred into the sample bottle and stored at −80 °C. Plasma TMAO, TMA and creatinine were determined by HPLC-MS/MS.

#### 2.4.2. Urine Sample Preparation

The 100 μL urine sample was added to 400 μL acetonitrile to precipitate the protein, and d9-TMAO with the final concentration of 2.0 μM was added to the urine sample as the internal standard. After the mixture was fully mixed, it was placed at −80 °C for 2 h and centrifuged for 15 min at 12,000× *g* at 4 °C. The supernatant was transferred into the sample bottle and stored at −80 °C. Urine TMAO and creatinine were determined by HPLC-MS/MS. Fractional excretion of TMAO was calculated as plasma TMAO/plasma creatinine × urine creatinine/urine TMAO × 100 [25].

#### 2.4.3. Cecum Sample Preparation

A mixture of 20 μL acetonitrile: methanol: water (40:40:20) was added to each milligram of cecal content, and d9-TMA with a final concentration of 2.0 μM was added to the cecal sample as the internal standard. After the mixture was fully mixed, it was placed at −80 °C for 2 h and centrifuged for 15 min at 12,000× *g* at 4 °C. The TMA in the supernatants was determined by HPLC-MS/MS [26].

#### 2.4.4. Determination of Enzymatic Activity of Hepatic FMOs

The enzymatic activity of hepatic FMOs was detected according to the previously reported method [25]. The specific operation was as follows: the mouse liver tissue was broken and homogenized in the RIPA lysate containing 1 mM PMSF, and when the liver tissue was lysed completely, the protein content was determined according to the operation instructions of the BCA protein quantitative kit (Biyuntian, Shanghai, China). The enzyme activity reaction experiment was then carried out, and the reaction system was 250 μL, including 1 mg liver protein homogenate, 100 μM d9-TMA, and 100 μM NADPH in 10 mM HEPES (pH 7.4). After incubation at 37 °C for 8 h, 0.2 N formic acid was added to terminate the reaction, then the filtered sample (ValueLab filter PTFE-Q 0.2 μm, Agilent) was transferred to the injection bottle and stored at −80 °C. The content of d9-TMAO was detected by HPLC-MS/MS.

#### 2.4.5. Detection of TMAO, TMA, Creatinine, d9-TMAO and d9-TMA by HPLC-MS/MS

Quantification of TMAO, TMA, creatinine, d9-TMAO and d9-TMA was performed using an Q Exactive Focus LCMS (Thermo Fisher Scientific, San Jose, CA, USA) [27]. Chromatographic separation was achieved using an Acquity UPLC BEH Amide (2.1 × 100 mm, 1.7 µm column) analytical column with gradient elution of solvent A (10 mM ammonium formate, pH 3.5) and solvent B (acetonitrile). The column was heated to 40 °C, autosampler and sample pan were maintained at 4 °C. The gradient was 5% A for 0.15 min, to 15% A in 1.2 min, to 20% A in 3 min, to 30% A in 6 min, to 45% A in 7 min, at 45% A for 4 min, to 5% A in 12 min, at 5% A for 3 min. The flow rate was 0.3 mL/min and the total run time was 15 min. 

The MS/MS detection was performed on a Q Exactive-Orbitrap mass spectrometer (Thermo Scientific, San Jose, CA, USA) equipped with a heated electrospray ionization (ESI) source. Analytes were detected in positive ionization mode using a parallel reaction monitor (PRM). The spray voltage was set to 3.5 kV and the capillary temperature was set to 320 °C. The ion transitions were m/z 76.07569 → 58.06588 for TMAO, (N) CE = 80, *m*/*z* 85.13218 → 66.11606 for d9-TMAO, (N) CE = 100, *m*/*z* 60.08078 for TMA, (N) CE = 10, *m*/*z* 69.13727 for d9-TMA, (N) CE = 10, and *m*/*z* 114.06619 → 72.04531 for creatinine, (N) CE = 60. Signal output was captured and processed with Xcaliber 2.2 SP1.48 (Thermo Fisher Scientific, San Jose, CA, USA).

TMA is an extremely volatile small molecule. It should be operated quickly in the process of sample preparation and detection, and the whole process was kept at low temperature. The samples were detected in random order to minimize errors due to processing time.

### 2.5. Determination of B. breve and B. longum Supplementation Effects on Gut Microbiota Composition 

The composition of the cecal microbial community was measured according to previously described methods [28]. Briefly, metagenomic DNA was extracted from frozen cecal samples using a Fast DNA SPIN Kit for Feces (MP Biomedicals, Santa Ana, CA, USA) according to the manufacturer’s instructions. The V3-V4 region of 16S rDNA genes was amplified as described previously [29]. All purified polymerase chain-reaction amplicons were mixed for sequencing using an Illumina Miseq platform (Illumina, CA, USA). The 16S rDNA sequence data were analyzed using Quantitative Insights Into Microbial Ecology (QIIME2, version 2019.7, https://view.qiime2.org/, accessed on 6 March 2022) [30]. The raw Illumina paired-end reads were screened after the removal of low-quality sequences (<30) and short sequences (<200 bp). The remaining sequences were spliced. The trimmed sequences with a >97% threshold of similarity were assigned to an operational taxonomic unit (OTU) for further analysis through online software. A pre-trained SILVA 16S rRNA classifier (silva-132-99-nb-341F-806R_classifier.qza) was used to perform the taxonomic classification. Alpha and beta diversity measures were calculated using the QIIME pipeline [30]. The functions of gut microbiota were predicted utilizing PICRUSt (v1.1.4, http://picrust.github.io/picrust/, accessed on 6 March 2022) [31]. 

### 2.6. Statistical Analysis

Data are reported as mean ± standard deviation (SD) with statistical significance inferred where *p* < 0.05. Statistical analysis was performed with one-way ANOVA with post hoc LSD test in SPSS software (version 22.0, Chicago, IL, USA) and GraphPad Prism (v6.0, GraphPad software, La Jolla, CA, USA). Graphs were obtained by GraphPad Prism software (v6.0, GraphPad software, La Jolla, CA, USA).

## 3. Results

### 3.1. Plasma TMAO Reached a Maximum Concentration 4 h after Fasting

**Figure 2:**
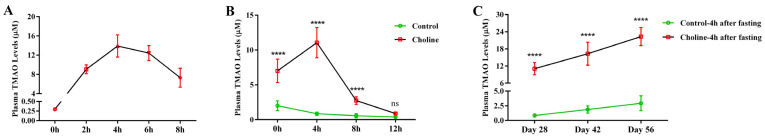
The change of plasma TMAO concentration after different treatments: (**A**) plasma TMAO concentration after intragastric administration of choline chloride, *X*-axis means different time point after gavage; (**B**) plasma TMAO concentration of different fasting time, *X*-axis means different time point after fasting; (**C**) plasma TMAO concentration of 4 h fasting at different feeding time. (**B**,**C**) **** *p* < 0.0001 versus the control group, “ns” means no significance.

After intragastric choline chloride administration, the concentration of TMAO in plasma rapidly increased from 0 to 4 h, reached the maximum at 4 h and then gradually decreased from 4 to 8 h (Figure 2A). Plasma TMAO concentrations were detected on day 28 after mice were fed with a standard control diet or 1.0% choline diet. It was found that plasma TMAO concentrations in mice fed with 1.0% choline diet varied with fasting time (Figure 2B, Supplementary Materials Figure S1A,B). In the control group fed the standard control diet (0.1% choline), the plasma TMAO concentration gradually decreased as the fasting time increased, whereas in the group fed the 1.0% choline diet, the plasma TMAO concentration increased for the first 4 h of fasting and then gradually decreased. The plasma TMAO concentration trends were similar among mice fed the 1.0% choline diet and those that received a single intragastric administration of choline chloride, with both reaching a peak at 4 h. Therefore, we selected the 4 h fasting time point to compare the effects of *Bifidobacterium* supplementation on plasma TMAO concentrations. The plasma TMAO concentration was measured after fasting for 4 h at 28 [26], 42 and 56 days after beginning the experiment. In mice in the control and 1.0% choline groups, the plasma TMAO concentrations increased over time. The percentage change from four to eight weeks was similar in these two groups (Figure 2C).

### 3.2. Choline Intake and B. breve and B. longum Supplementation Did Not Significantly Affect Blood Lipids

**Figure 3:**
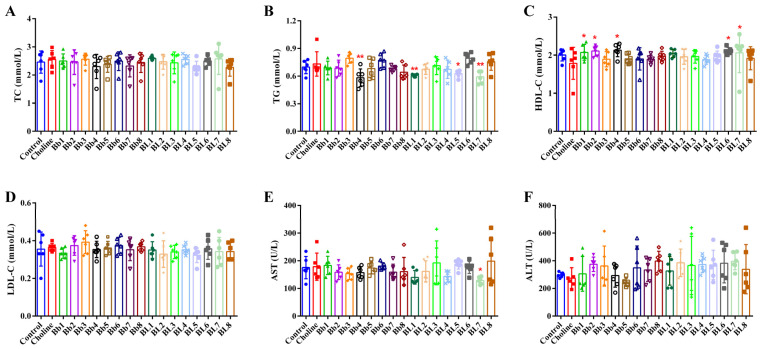
Plasma biochemical parameters: (**A**) total cholesterol level, (**B**) triglyceride level, (**C**) high-density lipoprotein-cholesterol level, (**D**) low-density lipoprotein-cholesterol level, (**E**) aspartate aminotransferase level, (**F**) alanine aminotransferase level. Values are mean ± SD; six mice per group. * *p* < 0.05, ** *p* < 0.01 versus the choline group.

The body weight of mice increased steadily before blood was collected from the orbital venous plexus. Blood collection caused a slight decrease in body weight in all groups of mice (Supplementary Materials Figure S2A). By treating the body weights as longitudinal data and some ad-hoc statistical analyses, we noted that the choline, Bb4, BL1, and BL7 groups, which were of interest in our study, had similar growth curves as the control group (Supplementary Materials and Methods 4 and Table S2). *B. breve* and *B. longum* supplementation had no significant effect on body weight trend and organ indexes after choline treatment (Supplementary Materials Figure S2). No significant differences in TC, TG, HDL-C and LDL-C concentrations between the choline and control groups were detected (Figure 3A–D). *B. breve* and *B. longum* supplementation could affect these parameters. Among them, Bb4, BL1, BL5 and BL7 significantly reduced plasma TG concentrations, and Bb1, Bb2, Bb4, BL6 and BL7 significantly increased plasma HDL-C concentrations. AST and ALT are indicators that reflect the degree of liver damage in mice. Figure 3E,F both show no significant differences in AST and ALT concentrations between the choline group and the control group, indicating that choline feed did not damage the liver. In groups supplemented with *B. breve* and *B. longum*, strains had no significant effects on ALT. Only BL7 significantly reduced plasma AST concentrations. 

### 3.3. Bb4, BL1 and BL7 Supplementation Significantly Decreased Plasma TMAO, Plasma TMA and Cecal TMA Concentrations

**Figure 4:**
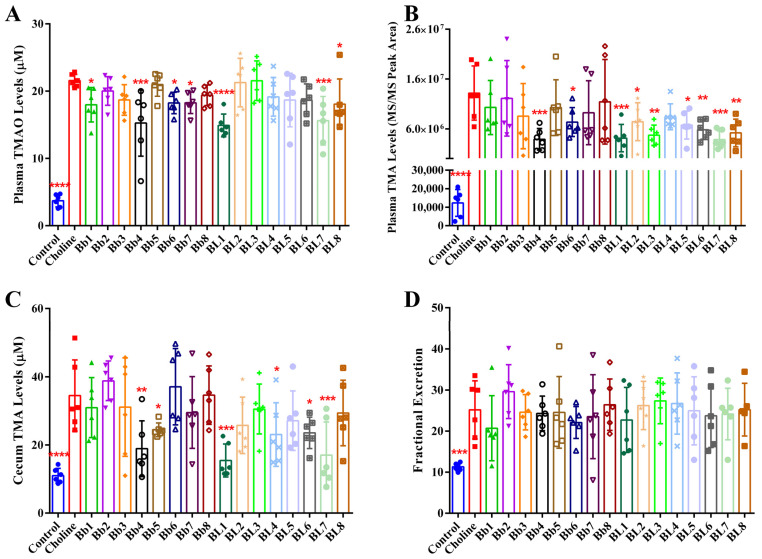
Some *Bifidobacterium* strains decreased the levels of plasma TMAO, plasma TMA and cecal TMA in mice to some extent: levels of (**A**) plasma TMAO, (**B**) plasma TMA, (**C**) cecal TMA; (**D**) fractional excretion of TMAO. Values are mean ± SD, six mice per group. * *p* < 0.05, ** *p* < 0.01, *** *p* < 0.001, **** *p* < 0.0001 versus the choline group.

In the previous experiment, as shown in Figure 2A,B, the plasma TMAO concentrations in mice reached its peak at 4 h after intragastric administration of choline chloride or fasting. Therefore, we compared the effects of different *Bifidobacterium* strains on plasma TMAO concentrations at 4 h after fasting. As shown in Figure 4A, the plasma TMAO concentration in the choline group was significantly higher than in the control group at 4 h after fasting at 42 days. Compared with the choline group, Bb1, Bb4, Bb6, Bb7, BL1, BL7 and BL8 significantly reduced plasma TMAO concentrations. Among them, the proportion of Bb4, BL1 and BL7 reducing plasma TMAO were the largest, which were 29.22%, 30.89% and 27.60%, respectively. The plasma TMA concentration was also significantly higher in the choline group than that in the control group (Figure 4B). Compared with the choline group, Bb4, Bb6, BL1, BL2, BL3, BL5, BL6, BL7 and BL8 significantly reduced plasma TMA concentrations. Among them, Bb4, BL1 and BL7 showed the largest proportion of reducing plasma TMA, which are 70.50%, 68.70% and 71.04%, respectively. Moreover, the cecal TMA concentrations of the choline group were significantly higher than that in the control group (Figure 4C). Compared with the choline group, Bb4, Bb5, BL1, BL4, BL6 and BL7 significantly reduced cecal TMA concentrations. Among them, Bb4, BL1 and BL7 had the top proportion of reducing cecal TMA, which were 45.14%, 55.18% and 50.68%, respectively. TMAO is mainly excreted through the kidney [32,33], and measuring TMAO fractional excretion showed that the fractional excretion of TMAO in the choline group was significantly higher than in the control group (Figure 4D). *Bifidobacterium* supplementation had no effect on the fractional excretion of TMAO. These findings indicate that Bb4, BL1 and BL7 significantly reduce plasma TMAO, plasma TMA and cecal TMA concentrations in mice, but not via the increased fractional excretion of TMAO. These strains have been preserved at the Food Biotechnology Centre of Jiangnan University under the respective serial numbers CCFM1217, CCFM1218 and CCFM1216.

### 3.4. B. breve and B. longum Supplementation Did Not Affect FMO3 and FXR Protein Expression, or Hepatic FMO Enzyme Activities

**Figure 5:**
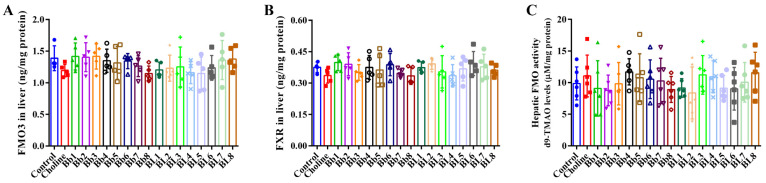
*Bifidobacterium* did not affect the expression levels of FMO3 and FXR and the enzyme activity of hepatic FMOs: (**A**) FMO3 in liver, (**B**) FXR in liver, (**C**) hepatic FMO activity. Values are mean ± SD, six mice per group.

Hepatic FMO3 plays an important role in the synthesis of TMAO from TMA; the overexpression of hepatic FMO3 significantly increases plasma TMAO concentrations. FMO3 expression is affected by dietary bile acids, including the bile acid-activated nuclear FXR [25]. No differences in FMO3 and FXR protein expression were observed between the choline and control groups (Figure 5A,B). *Bifidobacterium* supplementation did not significantly change the protein expression of FMO3 and FXR. Similarly, no significant differences in hepatic FMO activities were detected between the choline and control groups (Figure 5C). Compared with the choline group, *Bifidobacterium* supplementation slightly affected FMO activities, but the differences were not significant.

### 3.5. Bb4, BL1, BL7 and BL8 Supplementation Significantly Decreased the Protein Levels of Cecum CutC and CutD 

**Figure 6:**
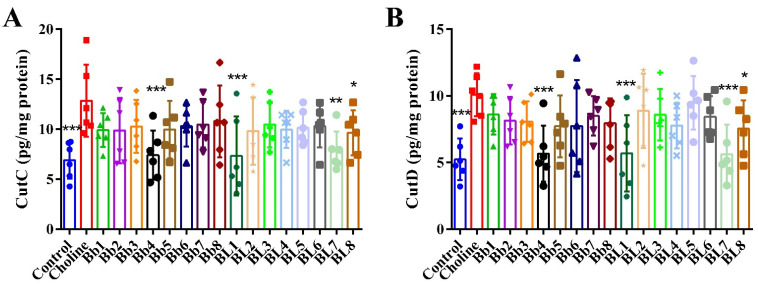
Some *Bifidobacterium* strains decreased the protein levels of cecum CutC and CutD in mice to some extent: (**A**) CutC levels in cecum, (**B**) CutD levels in cecum. Values are mean ± SD, six mice per group. * *p* < 0.05, ** *p* < 0.01, *** *p* < 0.001 versus the choline group.

As shown in Figure 6A, the protein level of cecum CutC in the choline group was significantly higher than that in the control group. Compared with the choline group, Bb4, BL1, BL7 and BL8 significantly reduced the protein levels of cecum CutC. The expression level of cecum CutD was also significantly higher in the choline group than in the control group (Figure 6B). Compared with the choline group, Bb4, BL1, BL7 and BL8 significantly reduced the levels of cecum CutD in the mice of these groups.

### 3.6. B. breve and B. longum Supplementation Alleviated Choline-Induced Gut Microbiota Dysbiosis 

**Figure 7:**
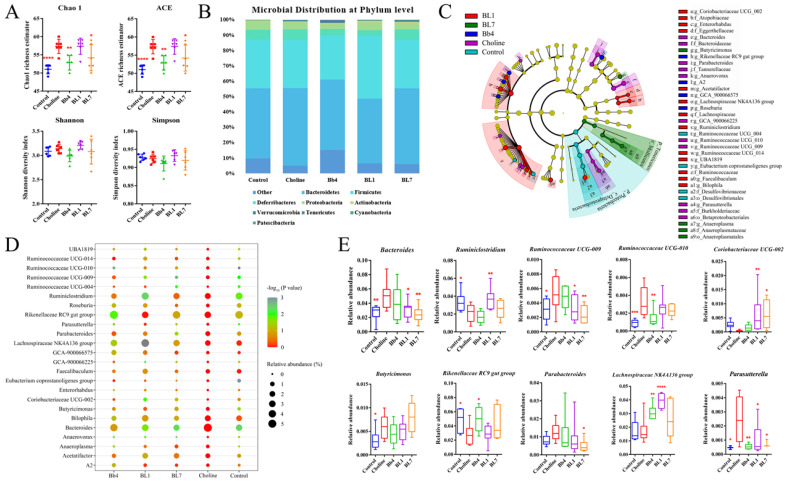
*Bifidobacterium* modulated the composition of gut microbiota: (**A**) α diversity of cecal microbial, (**B**) microbial distribution at phylum level; (**C**) plot LEfSe cladogram of cecal microbial, LDA > 2, *p* < 0.05; (**D**) Pretty Heatmap of cecal microbial, *X*-axis means the group, *Y*-axis means the genus, the color means the significance (*p* value), and the circle size means the relative abundance; (**E**) relative abundance of the genus level. Values are mean ± SD, six mice per group. * *p* < 0.05, ** *p* < 0.01, *** *p* < 0.001, **** *p* < 0.0001 versus the choline group.

Increases in plasma TMAO concentrations induced by a high choline diet are closely related to gut microbiota composition [4,5]. To explore the effects of *B. breve* and *B. longum* supplementation on the gut microbiota of choline-fed mice, we determined the cecal microbial composition by 16S rDNA sequencing. In terms of α-diversity, the chao1 and ACE indices of the choline group were significantly increased compared with the control group (Figure 7A). The chao1 and ACE indices of the Bb4- and BL1-supplemented groups were similar to those of the control group and significantly reduced compared with the choline group. BL7 supplementation did not significantly affect the diversity parameters. No significant differences in the Shannon and Simpson indices were detected between the control, choline and *Bifidobacterium*-supplemented groups. In addition, a principal coordinates analysis of the microbial taxa revealed that *B. breve* and *B. longum* supplementation had no significant effect on microbial composition between groups (Supplementary Material Figure S3A). 

As shown in Figure 7B and Figure S3B, the phyla with higher relative abundance, namely, Bacteroidetes, Firmicutes, Deferribacteres, Proteobacteria and Actinobacteria, remained the same across all of the experimental groups. Compared with the choline group, *B. breve* and *B. longum* supplementation affected the phylum-level relative abundance to varying degrees, and the relative abundance of Firmicutes was significantly higher in the BL1 group. At the genus level (Figure 7E), linear discriminant analysis effect size (LEfSe) and Pretty Heatmap (Figure 7C,D and Figure S3C) showed that *Bacteroides*, *Ruminococcaceae* UCG-009, *Ruminococcaceae* UCG-010, *Butyricimonas* and *Parasutterella* were significantly increased, and *Ruminiclostridium* and the *Rikenellaceae* RC9 gut group were significantly decreased in the choline group, compared with the control group. *B. breve* and *B. longum* supplementation changed the relative abundance of certain genera. Compared with the choline group, *Bacteroides* and *Ruminococcaceae* UCG-009 were significantly decreased, and *Coriobacteriaceae* UCG-002 was significantly increased, in the BL1- and BL7-supplemented groups. The relative abundance of *Ruminiclostridium* was significantly higher in the BL1 group. In the Bb4 group, *Ruminococcaceae* UCG-010 significantly decreased and the *Rikenellaceae* RC9 gut group significantly increased. *Parabacteroides* significantly decreased in the BL7 group. The *Lachnospiraceae* NK4A136 group significantly increased in the Bb4 and BL1 groups and *Parasutterella* significantly decreased in the Bb4, BL1 and BL7 groups.

**Figure 8:**
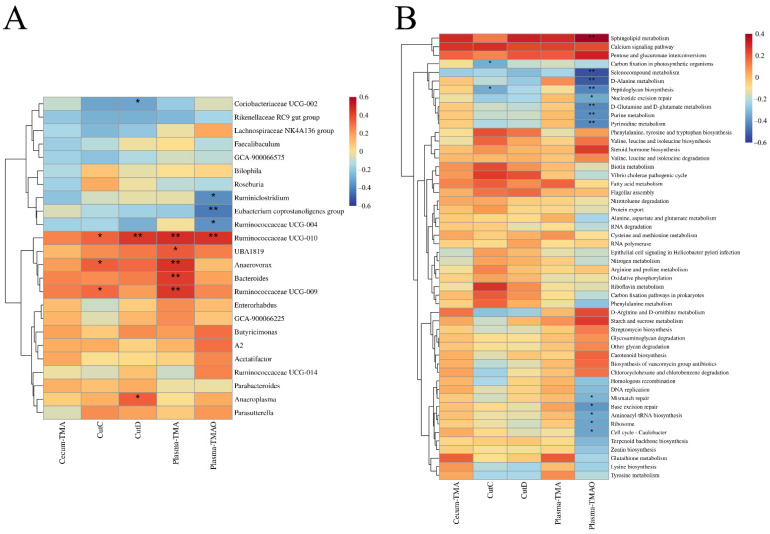
*Bifidobacterium* remodeled the structure of the gut microbiota then regulated the potential function of the gut microbiota: (**A**) correlation among plasma TMAO, plasma TMA, cecum TMA, the expression levels of cecum CutC and CutD and genus with significant differences; (**B**) correlation between plasma TMAO, plasma TMA, cecum TMA, the expression levels of cecum CutC and CutD and the pathway/function with significant differences. * *p* < 0.05, ** *p* < 0.01.

Furthermore, Pearson’s correlation analysis revealed that the abundance of some genera was significantly related to plasma TMAO and TMA concentrations, the expression levels of cecum CutC and CutD (Figure 8A). *Ruminococcaceae* UCG-010 was significantly positively correlated with plasma TMAO concentrations, and the *Eubacterium coprostanoligenes* group, *Ruminiclostridium* and *Ruminococcaceae* UCG-004 were significantly negatively correlated with plasma TMAO concentrations. *Ruminococcaceae* UCG-010, *Anaerovorax*, *Bacteroides* and *Ruminococcaceae* UCG-009 and UBA1819 were significantly positively correlated with plasma TMA concentrations. Furthermore, *Ruminococcaceae* UCG-010 and *Anaeroplasma* were significantly positively correlated with the expression levels of cecum CutD, while Coriobacteriaceae UCG-002 was significantly negatively correlated with the expression levels of cecum CutD. *Ruminococcaceae* UCG-010, *Anaerovorax* and *Ruminococcaceae* UCG-009 were significantly positively correlated with the expression levels of cecum CutC. In general, these results indicated that *B. longum* and *B. breve* supplementation affected the abundance of specific microbial taxa. Changes in the composition of the gut microbiota may relate to the decrease in plasma TMAO, plasma TMA, cecum TMA concentrations and the expression levels of cecum CutC and CutD. 

The function of cecal microbiota was predicted using PICTRUSt2 and the Kyoto Encyclopedia of Genes and Genomes (KEGG) pathway/function with significant differences screened, based on the LEfSe results (Figure S4). Pearson’s correlation analysis was used to detect significant relationships among pathway/function, plasma TMAO, plasma and cecal TMA concentrations, the expression levels of cecum CutC and CutD. Sphingolipid metabolism was significantly positively correlated with plasma TMAO concentration. Selenium metabolism, D-alanine metabolism, peptidoglycan biosynthesis, D-glutamine and D-glutamate metabolism, purine metabolism, pyrimidine metabolism, nuclear excision repair, base excision repair, ribosome, aminoacyl tRNA biosynthesis, mismatch repair and cell cycle–*Caulobacter* were significantly negatively correlated with plasma TMAO concentration. Carbon fixation in photosynthetic organisms and peptidoglycan biosynthesis were significantly negatively correlated with the expression level of cecum CutC (Figure 8B). *B. longum* and *B. breve* supplementation could, therefore, possibly remodel the structure of the gut microbiota and regulate gut microbiota functions (Supplementary Material Figure S3D).

## 4. Discussion

Choline is a nitrogen-containing compound and an essential micronutrient for the human body. Choline plays a role in human metabolism and participates in cell membrane functions, methyl transport and neurotransmission [34,35]. Choline is mainly absorbed from the diet but can also be obtained via de novo synthesis [34]. Some bacteria catalyze choline into TMA. In humans and other vertebrates, dietary choline is transformed into TMA only by intestinal symbionts [36]. In humans, TMA is then rapidly oxidized to TMAO by liver FMO3 [37]. This microbe-dependent host metabolic response has been linked to a variety of diseases, including inherited metabolic disease, trimethylamine (fish malodor syndrome) [38], non-alcoholic fatty liver disease [39] and, most recently, respiratory and cardiovascular diseases [4]. Dietary composition affects the community structure of intestinal microorganisms [40,41]. Probiotics are living microorganisms that are beneficial to humans and have great potential for reshaping the gut microbiota structure [42,43]. Nowadays, emerging evidence suggests that it is likely that toxic TMA, not TMAO, is harmful to the human body [44]. Probiotics that effectively reduce plasma TMAO, plasma TMA and cecal TMA concentrations may provide an effective and safe approach to preventing AS and reducing the toxicity caused by TMA. We supplemented choline-fed mice with different *Bifidobacterium* strains based on the choline/TMA/TMAO pathway and found that some strains significantly reduced plasma TMAO and plasma and cecal TMA concentrations.

Previous studies have reported a dose-dependent relationship between plasma TMAO concentrations and AS [4]. Moreover, increases in plasma TMAO concentration caused by a high choline diet are closely related to the gut microbiota composition [4]. Our results showed that plasma TMAO concentrations in choline-treated mice peaked at 4 h after fasting and continuously accumulated in the body upon extended feeding with a choline-enriched diet. As a result of weekly orbital venous plexus blood collection starting from week 3, the mice showed a trend of decreasing body weight. In order to avoid false positive results caused by weight loss, an ad hoc statistical analysis of the body weight was performed. From Table S2, it could be found that most of the treatment groups, including the choline, Bb4, BL1, and BL7 treatments which were of interest in this study, had similar growth curves to the control group. This suggests that the physiological effects of blood collection were similar in each group. Probiotic interventions could alleviate plasma TMAO, and plasma and cecal TMA concentrations, to varying degrees. These changes appear to be related to the changes in the gut microbiota, as FMO3 and FXR protein expression, hepatic FMO enzyme activities and TMAO fractional excretion were unaffected by *Bifidobacterium* supplementation. Moreover, the remission ability was strain-specific. This has also been reported in some previous studies. Ramireddy et al. [45] found *L. plantarum* LP1145, *L. amylovorus* LAM1345 and mixed probiotics could significantly reduce the levels of serum TMAO and TMA. Chen et al. [18] also found that plasma TMAO concentration peaked 4 h after administration of a single choline dose. In this case, resveratrol reduced TMA production by inhibiting the metabolism of gut microbiota, which reduced plasma TMAO synthesis and subsequently prevented the occurrence of AS. *Lactobacillus plantarum* ZDY04 similarly reduces the production of TMAO via gut microbiota modulation and did not affect liver FMO3 [26]. *Enterbacter aerogenes* ZDY01, which reduced TMA in vitro, reduced plasma TMAO by changing gut microbiota [46].

Craciun and Balskus [47] identified a choline utilization gene cluster in *Desulfovibrio*. Among them, a glycyl radical enzyme (*cutC*) and a glycyl radical-activating protein (*cutD*) were found to play critical roles in choline metabolism. Glycyl radical enzymes are widespread in anaerobic microorganisms. Members of this enzyme family use free radical intermediates derived from highly active proteins to promote chemical reactions such as C–C bond formation, C–C bond cleavage and dehydration. The C–N bond lyase, which is a glycine free radical enzyme involved in choline metabolism, is also a completely unique active function of this family [48]. In a previous study, 89 bacterial genomes containing *cut* gene clusters were identified by searching for *cutC* homologues in the National Center for Biotechnology Information (NCBI) database, including organisms related to choline degradation (*Desulfovibrio*, *Clostridium*, *Streptococcus*, *Klebsiella* and *Proteus*) and some genera that were not known to be involved in choline degradation. In particular, choline utilization did not appear to be evenly distributed among the bacterial phyla of the human intestine. For example, *cut* gene clusters were found in Firmicutes, Proteobacteria and Actinobacteria, but were completely absent in Bacteroidetes, despite it being the predominant component of the intestinal microbiota [47]. In this study, the relative abundance of *Ruminococcaceae* UCG-009 and UCG-010, which belong to the Firmicutes, significantly increased after choline treatment and were significantly positively correlated with plasma TMAO, plasma TMA concentration and the expression levels of cecum CutC and CutD. Bb4, BL1 and BL7 supplementation significantly reduced the relative abundance of these two genera, which may reduce *cut* gene clusters, leading to a decrease in TMA and TMAO. Additionally, it was reported that *Ruminococcaceae* members had the ability to degrade plant polysaccharides to glucose and short-chain fatty acid, which stimulated the production of glucose and hepatic triglyceride, thus increasing circulation glucose and lipids [49]. *Ruminococcaceae* family was positively correlated with atherosclerosis lesion size in apoE^-/-^ mice [50], and was also positively correlated to blood IgM levels and colitis [51]. Therefore, the reduction in *Ruminococcaceae* by Bb4, BL1 and BL7 supplementation may reduce *cut* gene clusters and thus intestinal TMA and plasma TMAO on the one hand, and the circulation glucose, lipids and inflammation on the other hand.

*Bacteroides*, regarded as potential pathogens for CVDs, are highly abundant in the gut microbiota of people with cardiac valve calibration and coronary artery disease, and are associated with dyslipidemia [52]. In this study, the relative abundance of *Bacteroides* significantly increased after choline treatment, and there was a positive correlation between this bacteria and plasma TMA. Although *cut* gene has been reported to be completely absent in Bacteroidetes, some clinical trials have also reported the change in *Bacteroides* in cardiovascular patients [53]. Taken together with our results, this suggests that *Bacteroides* may affect TMA production through some other mechanism. The ability of BL1 and BL7 to reduce plasma TMA may partly benefit from their regulation of *Bacteroides* abundance. *Ruminiclostridium* possessed strong affinity with polysaccharide β-Glucan [54], which may promote the production of short chain fatty acids (SCFAs). SCFAs are generally believed to be beneficial to the human body. The decrease in *Ruminiclostridium* caused by choline may be indicative of i562-563ts adverse effects on the body. Although one strain recovered the relative abundance of *Ruminiclostridium* significantly, change in abundance was not significantly correlated with plasma TMAO and plasma TMA concentrations. 

Through a functional prediction analysis of cecal samples, we found that sphingolipid metabolism increased significantly after choline treatment compared with the control group and was significantly positively correlated with plasma TMAO concentrations. Sphingolipids plays a very important role in cell membrane formation, signal transduction, and plasma lipoprotein metabolism [55]. It was reported that sphingomyelin can be catalyzed by sphingomyelinase to generate the second messenger-like molecule ceramide, activating the tumor necrosis factor signaling route and further inducing nuclear transcription factor kappa B activity [56]. BL1 and BL7 intervention attenuated the increase in sphingolipid metabolism, which indicated the possibility of a reduction in inflammation by these two strains.

Choline and TMA are small molecules that play an extremely important role in human life [35]. Choline is utilized as an energy source by microorganisms in the human gastrointestinal tract and other anaerobic environments. During this process, the metabolite TMA is generated by microorganisms [57]. TMA is also a carbon source for bacteria and is transformed into methane by archaea in various marine environments [58]. In the human body, redundant TMA is oxidized into TMAO via liver FMOs, eventually increases plasma TMAO concentrations and causes a variety of diseases [4]. As an essential micronutrient, choline participates in cell membrane signals related to phospholipids, lipid transport by lipoproteins, methyl metabolism through the reduction in homocysteine, and neurotransmitter synthesis via acetylcholine [34,35]. However, choline treatment significantly reduces the metabolism of certain amino acids, peptidoglycans and nucleotides, and gene repair function, which may be due to excessive plasma TMAO concentrations. In this study, Bb4, BL1 and BL7 interventions significantly reduced plasma TMAO concentrations, thus potentially alleviating the disruption of physiological functions induced by excess choline. Analysis revealed that this may be related to its regulation of the gut microbiota. 

## 5. Conclusions

Long-term consumption of a choline-rich diet may result in TMAO accumulation in the plasma, which could increase the risk of AS. Choline intake did not affect the body weight change trend and organ indices of mice. Supplementation with certain *B. longum* and *B. breve* strains reduced plasma TMAO and TMA, cecal TMA concentrations, and the expression levels of cecum CutC and CutD to varying degrees without affecting FMO3 and FXR protein expression, the enzymatic activities of hepatic FMOs or TMAO fractional excretion. The *Bifidobacterium* strains that effectively reduced TMAO concentrations were found to significantly reduce the relative abundance of *Ruminococcaceae* UCG-009 and UCG-010 (Firmicutes), which belong to the Firmicutes with *cut* gene clusters. In addition, a reduction in *Ruminococcaceae* indicates a reduction in circulating glucose and lipids, which may be another pathway by which *Bifidobacterium* strains relate to the reduction of risk caused by excess choline. Although there is no evidence for the presence of the *cut* gene in *Bacteroides*, the effect of *Bifidobacterium* strains on *Bacteroides* also suggests a relationship between the abundance of this genus and TMA concentrations in the gut. Those effective *Bifidobacterium* strains were also found to significantly reduce sphingolipid metabolism, which may relate to a reduction in inflammation. The remodeling of the gut microbiota by these *Bifidobacterium* strains further alleviated the damage to physiological functions caused by choline treatment. Therefore, these *Bifidobacterium* strains may have therapeutic potential for alleviating TMAO-related diseases.

## Data Availability

The study did not report any data.

## Supplementary Materials

The following supporting information can be downloaded at https://www.mdpi.com/article/10.3390/nu14061222/s1. Figure S1: The change of plasma TMAO concentration after different treatments: (A) plasma TMAO concentration of mice treated with control diet at different time point after fasting, (B) plasma TMAO concentration of mice treated with 1.0% choline diet at different time point after fasting, **** *p* < 0.0001 versus 0 h, # *p* < 0.05, #### *p* < 0.0001 versus 4 h, and *p* < 0.05 versus 8 h. Figure S2: Average body weight and organ indices: (A) average body weight, (B) liver index, (C) kidney index, (D) spleen index, values are mean ± SD, six mice per group. Figure S3: *Bifidobac**terium* modulated the gut microbiota: (A) principal coordinates analysis of microbial taxa, (B) microbial distribution at phylum level, (C) plot LEfSe Results of cecal microbial, LDA > 2, *p* < 0.05, (D) relative abundance of the significant pathway/function, values are mean ± SD, six mice per group, * *p* < 0.05, ** *p* < 0.01, *** *p* < 0.001, **** *p* < 0.0001 versus the choline group. Figure S4: Plot LEfSe results of the significant pathway/function: LDA > 2, *p* < 0.05. Table S1: *Bifidobacteria* used in this study. Table S2: *p*-values of the likelihood ratio test statistics for testing whether the growth curves are similar among the treatment and control groups.
