# Supplementary material for: Bifidobacterium breve and Bifidobacterium longum Attenuate Choline-Induced Plasma Trimethylamine N-Oxide Production by Modulating Gut Microbiota in Mice
Source: Nutrients. 2022 Mar 14;14(6):1222. doi: 10.3390/nu14061222 (PMC8950610; doi:10.3390/nu14061222)
Supplement: Supplementary file 1 [file nutrients-14-01222-s001.zip › nutrients-1556429-supplementary.pdf]

Supplementary Materials and Methods

1 Strain identification

The strains were identified through the following steps：

(1) 16S rDNA PCR:

A. Bacterial 16S rDNA 50 μL PCR reaction system: 10×Taq buffer, 5 μL; dNTP, 5 μL; 27F, 0.5 μL; 1492R, 0.5 μL; Taq enzyme, 0.5 μL; template, 0.5 μL; ddH2O, 38 μL.

B. PCR conditions: 95 °C 5 min; 95 °C 10 s; 55 °C 30 s; 72 °C 30 s; step2-4 30×; 72 °C 5 min; 12 °C 2 min;

C. PCR products were check with gel imaging and sent to a professional sequencing company for sequencing. BLAST was used to identify the obtained sequencing results in GenBank.

(2) Whole genome sequencing: The extracted whole genome was sent to a professional sequencing company, and the whole genome of the bacteria was sequenced by a second-generation sequencer. The obtained sequence results were analyzed in GenBank using BLAST.

2 Anaerobic conditions for culture

To maintain an anaerobic environment, strains were cultured in MRS medium supplemented with 0.05% cysteine in an anaerobic workstation (Electrotek 400TG workstation, Eletrotek, West Yorkshire, UK). Palladium is used as a catalyst to react the hydrogen in the cylinder with the oxygen in the air to form water, so as to achieve the anaerobic effect. The oxygen content in the anaerobic workstation is less than 1%.

3 Bacterial suspensions preparation

Bifidobacterium strains were activated for 3 generations at 3% inoculum (v / v) in MRS medium containing 0.05% cysteine. After the strain is activated, it is further cultivated in large quantities. *Bifidobacterium* were collected by centrifugation at 6000 × g for 15 min at 4 °C. The collected bacteria was washed three times with pre-cooled sterile normal saline (containing 0.05% cysteine) and resuspended in a small volume of pre-cooled 30% sucrose solution and stored at -80°C. At the same time, the amount of bacteria in the resuspension was evaluated by the plate colony technique. Before it was used for gavage, in order to eliminate the influence of residual sucrose on the bacterial suspension, the stains were washed three times with pre-cooled sterile normal saline (containing 0.05% cysteine), and then diluted with sterile normal saline to a viable count of 1×10^9 CFU/mL. Mice in the control group were treated with sterile saline without bifidobacteria.

4 Statistical analysis for the similarity of growth curves

To illustrate the similarity of growth curves in 18 groups, we treated the body weight observations as longitudinal data over weeks and performed a series of statistical analyses to test whether the growth (weight) curves were similar between treatment (Bb1-Bb8; BL1-BL8; Choline) and control groups.

Firstly, we presented graphs of the average of body weights in each group in Figure S2 (A). Intuitively, almost all the growth curves were parallel to some extent, which indicated that the growth process of mice might be similar in different groups.

Secondly, we fitted the longitudinal data using a quadratic regression model and further performed a likelihood ratio test to support our finding. As discussed in Section 6.2 on page 144, a widely used approach for describing the time-varying patterns of the growth curves is in terms of simple polynomial trends, for example, linear or quadratic trends. Since all the curves in Figure S2 (A) looked like a U-shape, we fitted the growth curve by a quadratic model to capture the quadratic trend for each group. Specifically, we merged body weights in one treatment group and those in the control group, and further adopted the following model to fit the pooled data:

$$\mathrm{E}(Y_{ijt}) = b_1 + b_2\mathrm{t} + b_3\mathrm{t}^2 + b_4 i + b_5\mathrm{i}\times\mathrm{t} + b_6\mathrm{i}^2\times\mathrm{t}^2,$$

where $\mathrm{i} = 0$ (1) represents the control (treatment) group, $\mathrm{t} = 0, \ldots, 6$ is the week index, $\{Y_{ijt}: \mathrm{j} = 1, \ldots, n_i\}$ are the observed body weights in the ith group at the tth week, and n_i is the number of mouse in the ith group. Under this model, testing whether the growth curves are parallel is equivalent to testing whether the coefficients b_5 and b_6 are equal to zero. To achieve this, we fitted models with and without time-group interactions to the pooled data respectively and performed a likelihood ratio test for the null hypothesis $H_0: b_5 = b_6 = 0$. This can be implemented by using the gls() function with unstructured covariance in the R software.

Calibrated by the chi-sqaure distribution with 2 degrees of freedom, we reported the p-values corresponding to the likelihood ratio test statistics in Table S2. If the p-value is great than 0.05, we do not have enough evidence to show that the growth curves change differently over time between the treatment and control groups. From this point of view, we

could see that most of treatment groups, including the Choline, Bb4, BL1, and BL7 treatments which are of interest in our study, had similar growth curves as the control group.

Table S1. *Bifidobacteria* used in this study

Serial Number	Species	Original number	Sample
Bb1	*Bifidobacterium breve*	FZJHZD20M12[22]	Human faeces
Bb2	*Bifidobacterium breve*	FFJXM1M3	Human faeces
Bb3	*Bifidobacterium breve*	FJSWX17M1[22]	Human faeces
Bb4	*Bifidobacterium breve*	FFJND6M1[22]	Human faeces
Bb5	*Bifidobacterium breve*	FCJ1041[22]	Human faeces
Bb6	*Bifidobacterium breve*	FXJCJ32M7	Human faeces
Bb7	*Bifidobacterium breve*	FCQNA20M1	Human faeces
Bb8	*Bifidobacterium breve*	HuNan2016497	Human faeces
BL1	*Bifidobacterium longum*	HeNa13-5GMM	Human faeces
BL2	*Bifidobacterium longum*	FGDLZ4M1	Human faeces
BL3	*Bifidobacterium longum*	FJSNT53M9	Human faeces
BL4	*Bifidobacterium longum*	RG4-1[23]	Human faeces
BL5	*Bifidobacterium longum*	FGSZY6M4[23]	Human faeces
BL6	*Bifidobacterium longum*	HUB29-14	Human faeces
BL7	*Bifidobacterium longum*	M2-C-F01-14[24]	Human faeces
BL8	*Bifidobacterium longum*	FGXBM15M1	Human faeces

Table S2. *P*-values of the likelihood ratio test statistics for testing whether the growth curves are similar among the treatment and control groups.

Treatment	**Bb1**	**Bb2**	**Bb3**	**Bb4**	**Bb5**	**Bb6**	**Bb7**	**Bb8**	**Choline**
***P*-value**	0.113	0.471	0.369	0.511	0.019	0.134	0.505	0.413	0.238
Treatment	**BL1**	**BL2**	**BL3**	**BL4**	**BL5**	**BL6**	**BL7**	**BL8**	
***P*-value**	0.209	0.333	0.472	0.222	0.124	0.072	0.939	0.030	

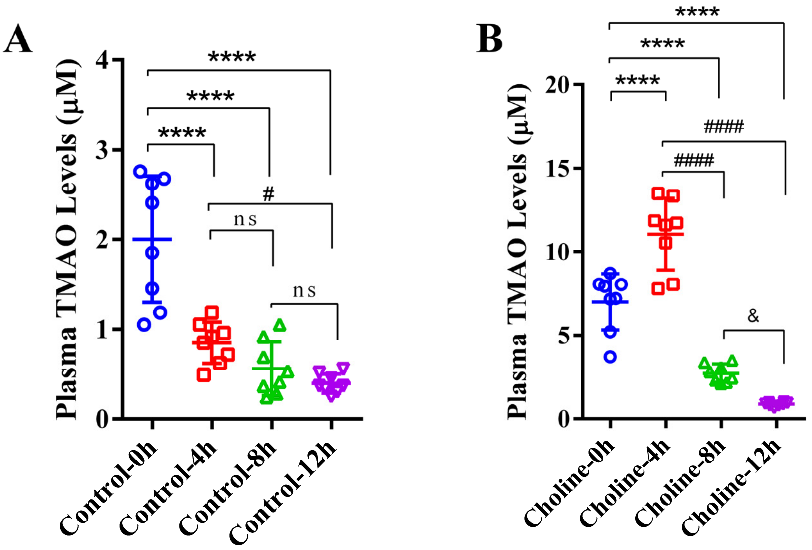

Figure S1. The change of plasma TMAO concentration after different treatments. (A) Plasma TMAO concentration of mice treated with control diet at different time point after fasting. (B) Plasma TMAO concentration of mice treated with 1.0% choline diet at different time point after fasting. **** $p < 0.0001$ versus 0h; # $p < 0.05$, #### $p < 0.0001$ versus 4 h; & $p < 0.05$ versus 8 h.

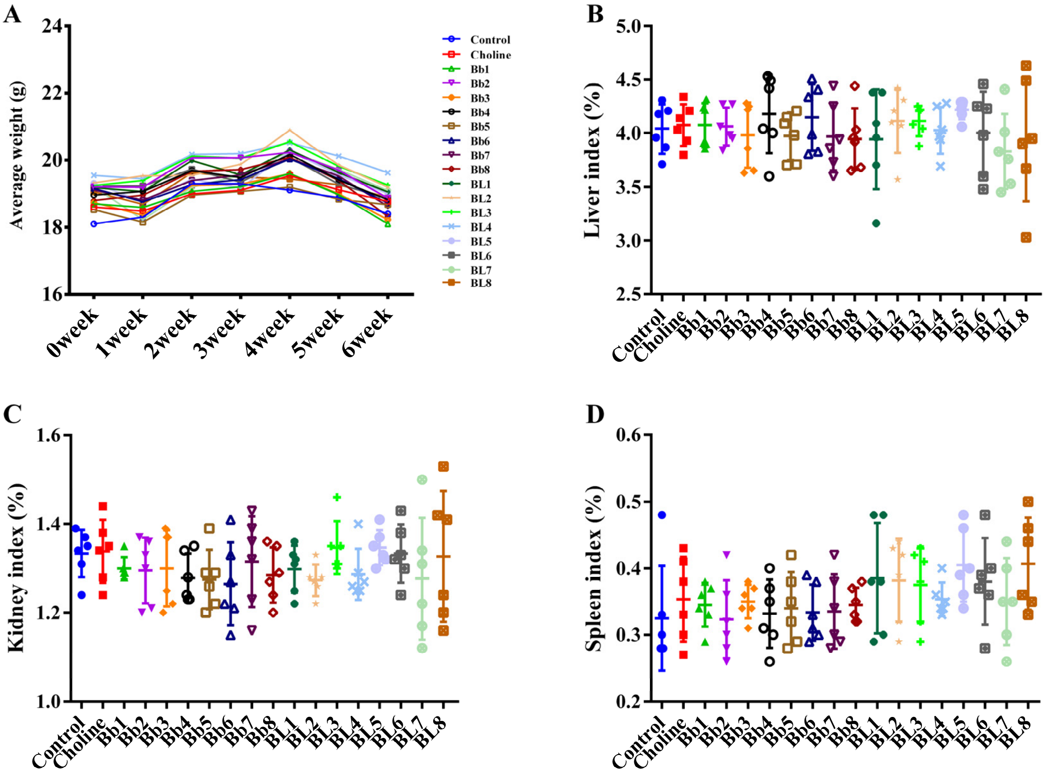

Figure S2. Average body weight and organ indices. (A) Average body weight. (B) Liver indice. (C) Kidney indice. (D) Spleen indice. Values are mean ± SD; six mice per group.

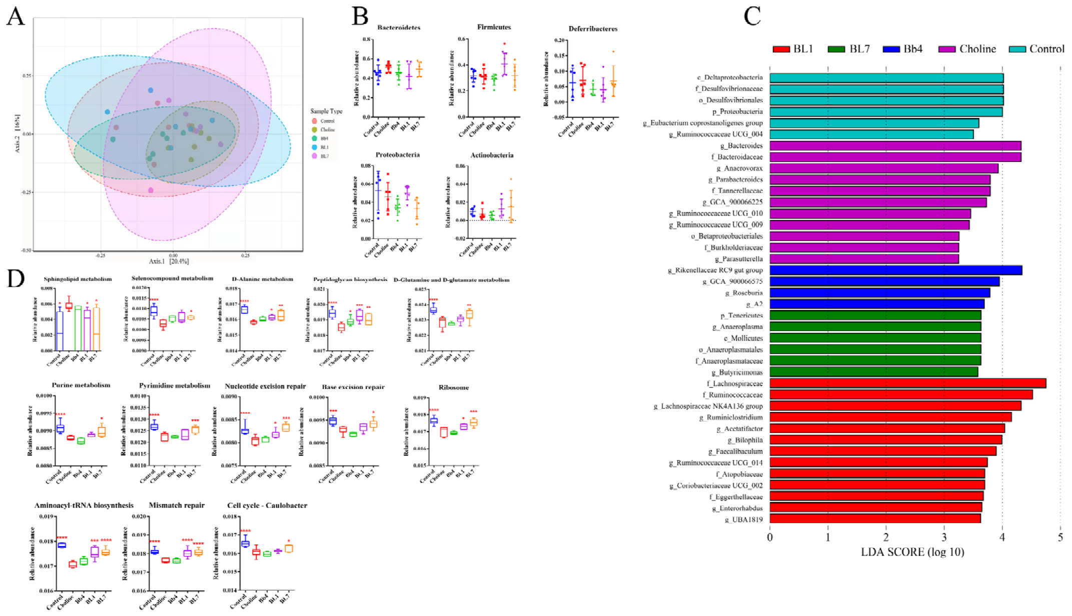

Figure S3. *Bifidobacterium* modulated the gut microbiota. (**A**) Principal coordinates analysis of microbial taxa. (**B**) Microbial distribution at phylum level. (**C**) Plot LEfSe Results of cecal microbial. LDA > 2, $p < 0.05$. (**D**) Relative abundance of the significant pathway/function. Values are mean ± SD, six mice per group. * $p < 0.05$, ** $p < 0.01$, *** $p < 0.001$, **** $p < 0.0001$ versus the choline group.

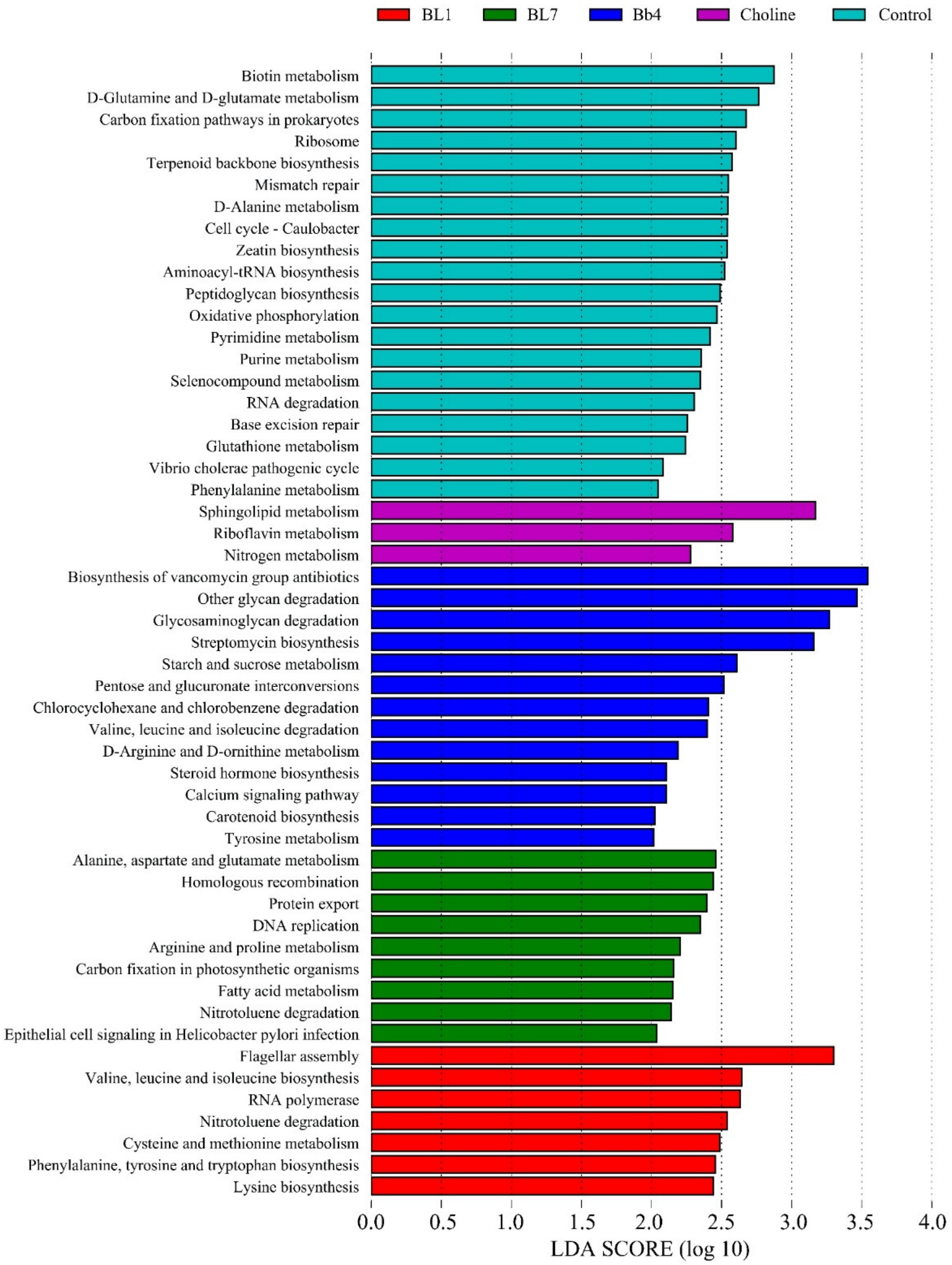

Figure S4. Plot LEfSe Results of the significant pathway/function. LDA > 2, $p < 0.05$.
